# Investigating the Role of Artemin and Its Cognate Receptor, GFRα3, in Osteoarthritis Pain

**DOI:** 10.3389/fnins.2022.738976

**Published:** 2022-01-27

**Authors:** Laura Minnema, Ankita Gupta, Santosh K. Mishra, B. Duncan X. Lascelles

**Affiliations:** ^1^Translational Research in Pain Program, Department of Clinical Sciences, College of Veterinary Medicine, North Carolina State University, Raleigh, NC, United States; ^2^Department of Clinical Sciences, College of Veterinary Medicine, North Carolina State University, Raleigh, NC, United States; ^3^Department of Molecular and Biomedical Sciences, College of Veterinary Medicine, North Carolina State University, Raleigh, NC, United States; ^4^Comparative Pain Research and Education Center, North Carolina State University, Raleigh, NC, United States; ^5^Thurston Arthritis Center, UNC School of Medicine, Chapel Hill, NC, United States; ^6^Department of Anesthesiology, Center for Translational Pain Research, Duke University, Durham, NC, United States

**Keywords:** pain, osteoarthritis (OA), artemin, GFRα3, TRPV1, monoiodoacetate (MIA)

## Abstract

Osteoarthritis (OA) associated pain (OA-pain) is a significant global problem. OA-pain limits limb use and mobility and is associated with widespread sensitivity. Therapeutic options are limited, and the available options are often associated with adverse effects. The lack of therapeutic options is partly due to a lack of understanding of clinically relevant underlying neural mechanisms of OA-pain. In previous work in naturally occurring OA-pain in dogs, we identified potential signaling molecules (artemin/GFRα3) that were upregulated. Here, we use multiple approaches, including cellular, mouse genetic, immunological suppression in a mouse model of OA, and clinically relevant measures of sensitivity and limb use to explore the functional role of artemin/GFRα3 signaling in OA-pain. We found the monoiodoacetate (MIA)-induced OA-pain in mice is associated with decreased limb use and hypersensitivity. Exogenous artemin induces mechanical, heat, and cold hypersensitivity, and systemic intraperitoneal anti-artemin monoclonal antibody administration reverses this hypersensitivity and restores limb use in mice with MIA-induced OA-pain. An artemin receptor GFRα3 expression is increased in sensory neurons in the MIA model. Our results provide a molecular basis of arthritis pain linked with artemin/GFRα3 signaling and indicate that further work is warranted to investigate the neuronal plasticity and the pathways that drive pain in OA.

## Introduction

While the term “arthritis” encompasses around 100 different types of joint disease, osteoarthritis (OA) is one of the most common forms of degenerative joint disease in humans and companion animals. OA’s prevalence is around 35.5% worldwide (Neogi, 2013) and similar for companion dogs and cats (Lascelles et al., 2010, 2012; Wright et al., 2019). Pain associated with OA represents one of the major health burdens in the industrialized world (Neogi, 2013).

Osteoarthritis is multifactorial in terms of etiology, with numerous factors contributing to the disease. Most OA research has focused on the disease *per se* (Malfait et al., 2013), which is surprising since pain and disability are the primary symptoms for patients who suffer from OA. Ongoing chronic pain from affected joints has a significant impact on a patient’s quality of life (Hoogeboom et al., 2013; Clauw et al., 2019). There are few effective treatments for OA-associated pain (OA-pain), and these are often associated with dangerous side effects. Treatments include corticosteroids and non-steroidal anti-inflammatory drugs (NSAIDs). Corticosteroids can be effective at relieving joint pain. However, they are a short-term solution (Gossec and Dougados, 2004), and repeat injections raise concerns about systemic exposure and effects on joint cartilage. In comparison, NSAIDs are associated with severe gastrointestinal bleeding and an increased risk of heart attack or stroke (Varga et al., 2017). Currently, a novel drug class, anti-nerve growth factor (NGF) monoclonal antibodies (mAbs), is under development for OA-pain management (Dimitroulas et al., 2014; Bannwarth and Kostine, 2017; Dietz et al., 2021). However, anti-NGF mAbs also have safety concerns which include risk of transient paresthesia and dysthesias, more quickly progressing OA (∼1.5–3.0%), and, in a small number of patients, a specific type of OA progression, rapidly progressing OA type 1 and 2 (Wise et al., 2021). Anti-NGF’s adverse effects are dose-dependent, with lower doses being safer, but lower doses are also less effective for pain relief (Wise et al., 2021). In brief, problems associated with these therapeutics potentially limit the treatment for OA-pain. Hence, to develop alternative analgesic therapies, we need to better understand the molecular players involved in OA-pain.

Recently, we identified key molecules in the chronic OA-pain pathway: the neurotrophic factor artemin and its receptor, GFRα3. Artemin is a Glial cell line-Derived Neurotrophic Factor family member and appears to be an important contributor to persistent pain conditions such as migraine, burning mouth syndrome, cystitis, neuropathic cold pain, and inflammatory bone pain (Elitt et al., 2008; Forrest et al., 2014; Shinoda et al., 2015; Lippoldt et al., 2016; Shang et al., 2016; Nencini et al., 2019). We have found elevated serum artemin concentrations in humans and dogs (Minnema et al., 2020) and cats (unpublished data) with naturally occurring OA. Additionally, we have shown that synovial fluid and serum artemin concentrations are related to joint pain in the naturally occurring dog model of OA-pain (Minnema et al., 2020; Gupta et al., 2021). We have also identified significantly increased GFRα3 and transient receptor potential vanilloid subfamily-1 (TRPV1) receptor expression in dogs’ dorsal root ganglia (DRG) serving osteoarthritic joints compared to healthy dogs (Minnema et al., 2020). Overall, these observations in the naturally occurring OA dog (and cat) model suggest a possible role of artemin/GFRα3 in OA-pain. Additionally, the artemin/GFRα3 complex can directly/indirectly activate TRP channel expression and activity and perpetuate pain (Elitt et al., 2006; Ikeda-Miyagawa et al., 2015). These mechanisms appear to be partly responsible for the association between artemin and increased noxious heat sensitivity *via* TRP channels. Both TRPV1 and TRPM8 are shown to be co-expressed with GFRα3 in the DRG (Elitt et al., 2008; Goswami et al., 2014) and may serve as a downstream target of artemin/GFRα3. However, no comprehensive work has been performed in the clinically important condition of OA-pain to evaluate the role of artemin/GFRα3, or the relationship between artemin/GFRα3 and downstream TRP channel signaling and if inhibition of artemin signaling can provide analgesic relief in OA-pain.

To start exploring the functional role of artemin/GFRα3 we used a monoiodoacetate (MIA) mouse model of OA-pain. MIA is a glyceraldehyde-3-phosphatase dehydrogenase inhibitor. The MIA model has been extensively used in mice and rats and has been well-characterized for pain phenotypes since its first description in 1985 (Kalbhen, 1985). The MIA model has been shown to have OA disease characteristics and is associated with robust mechanical hypersensitivity and decreased limb use (Harvey and Dickenson, 2009; Ogbonna et al., 2013; Kuyinu et al., 2016; Yuan et al., 2018). However, there is disagreement about thermal hypersensitivity in the MIA model of OA-pain (Harvey and Dickenson, 2009; Yuan et al., 2018). To the best of our knowledge, no one report in mice has evaluated all the algoplastic changes to mechanical, heat, and cold stimuli and limb use associated with MIA-induced OA-pain. Thus, our goal was to evaluate changes in hypersensitivity and limb use in the MIA model of OA-pain and perform initial work exploring the role of artemin/GFRα3 in mitigating these changes. After confirming the development of heat, cold, and mechanical sensitivity and alterations in static limb use in the mouse MIA model of OA-pain, we also explored behavioral alterations in *Trpv1* mutant mice, as TRPV1 is likely a downstream target of artemin/GFRα3. We demonstrated changes in expression of GFRα3 in the MIA model, and found increased mechanical and thermal hypersensitivity induced by artemin. Further, an anti-artemin mAb attenuated hypersensitivity and limb disuse. In summary, our results point to a significant role of artemin/GFRα3 signaling in OA-pain.

## Materials and Methods

All experiments were performed under Institutional Animal Care and Use Committee approval (NC State IACUC #19-047B) and are in strict accordance with the National Institute for Health’s Guide for the Care and Use of Lab Animals.

### Mice

In all experiments, across all groups, aged-matched, 4- to 7-week-old, adult male C57BL6 mice (Jackson Labs) were used. For the assessment of analgesic effects of a systemic anti-artemin mAb on limb use, both male and female C57BL6 mice were used. The average mouse weight was 25 g and mice were housed in groups of two to four and kept on a 12-h light–dark cycle with lights off at 1800 h. *Trpv1* knockout (KO; Jackson Labs-Stock No: 003770) mice were on a C57BL6 background. All animals had *ad libitum* access to chow (Purina LabDiet 5001) and water. Sample sizes for each experiment are listed under the figure legends.

### Preparation and Injection of Monoiodoacetate

Intra-articular injection of MIA was performed according to the method published by Pitcher et al. (2016). Following pilot dose-determination experiments, MIA (Sigma-Aldrich, I2512) was dissolved in sterile 0.9% saline and volumes of 10 μl containing 1 mg MIA were injected intra-articularly (Ogbonna et al., 2013; Pitcher et al., 2016) using a zero-dead space syringe with a 33G needle and a depth stop set at 2 mm (Hamilton). Control mice received 10 μl of sterile 0.9% saline. Right stifles were used for all injections. Mice were anesthetized for the injection using an induction box and 4% isoflurane carried in oxygen and maintained under anesthesia using a nose-cone delivering 2.0% isoflurane carried in oxygen. The injection site (right stifle) was cleaned with 70% ethanol prior to the injection. To reduce inflammation and abrasions, we did not shave the injection area. Instead, the ethanol scrub was used to flatten and part the fur for visualization of the injection site. The joint space was identified by flexing the leg and using a transversely applied 27G needle to identify the location of the distal patella ligament (depression between the distal pole of the patella and the proximal part of the tibial crest) (Pitcher et al., 2016). Mice were under anesthesia for no longer than 5 min.

### Behavior

Researchers performing behavioral assays were blinded to treatment groups to minimize bias. Behavioral assays were conducted at the same time of day (afternoon) for each time point. Mice were acclimated to the testing environment and each piece of equipment for 10 min before each time point. All animals were habituated to the handling and restraint necessary for the performance of all tests. At each time point, behavioral assays were repeated five times with 3–5 min between each measurement, unless otherwise noted.

#### Evoked Pain Behavior

Mechanical sensitivity was measured using the Ugo Basile Dynamic Plantar Aesthesiometer, referred to as the electronic von Frey. A mechanical stimulus is delivered to the hind paw and the force at which mouse withdrew its paw was recorded. Heat sensitivity was tested using the plantar assay (Hargreaves apparatus, Ugo Basile). Mice were placed in testing chambers on a glass plate. An infrared light source was focused on the plantar surface of the hind paw and the time taken to withdraw from the heat stimulus was recorded (Mishra et al., 2011). For cold measurement, a dry ice method was used to deliver the cold stimulus to the glass underneath the hind paw and the latency was recorded (Brenner et al., 2015).

#### Incapacitance Meter to Measure Static Limb Use

Static weight-bearing was measured using a Static Hind limb Incapacitance Meter (SHIM) connected to a system 8000 MicroMeassurements tool (Williams et al., 2019), and also measured using the IITC Incapacitance Meter (IC Meter) (IITC, Life Science, Woodland Hills, CA, United States). For the SHIM, data were collected from mice as described previously (Williams et al., 2019). Data were retained if the animal stood still in a relaxed position, without noticeably shifting weight, lifting, offloading a limb, or turning the head. Each animal was tested until five appropriate trials were obtained, each 5 s in length. Hind limb distribution of weight was recorded using the StrainSmart software and transferred to excel data files for the SHIM. For the IITC IC Meter, mice were tested for incapacitance of the injected ipsilateral right leg and the contralateral control leg as previously described (Bove et al., 2003; Longobardi et al., 2017). Each subject was recorded for 5 s long and a total of 12 replicates. Following data collection, mice were placed back into their home cage.

Weight-bearing on the ipsilateral limb of interest was expressed as symmetry indices (SI) (Longobardi et al., 2017) and Delta:


$$S\,I=100*\frac{X\,\left(c\,o\,n\,t\,r\,o\,l\right)-X\,\left(i\,n\,j\,e\,c\,t\,e\,d\right)}{0.5^{*}\,\left(X\,\left(i\,n\,j\,e\,c\,t\,e\,d\right)+X\,\left(c\,o\,n\,t\,r\,o\,l\right)\right)}$$

Where X = weight placed on the limb; injected = MIA or saline injected limb; control = non-injected contralateral limb.


Delta⁢(grams)=Weight⁢placed⁢on⁢the⁢right⁢leg-Weig⁢ht⁢placed⁢on⁢the⁢left⁢leg

### Assessment of the Hyperalgesic Effects of Artemin

Naïve male C57BL6 mice received hind paw injections of artemin to determine whether artemin can induce localized hypersensitivity. A 200-ng injection of artemin (R&D Biosystems; Cat. 1085-AR/CF) was delivered in 10 μl of sterile 0.9% saline using an insulin syringe to the subcutaneous layer of the left hind paw while mice were conscious and gently restrained. Control mice received 10 μl of sterile 0.9% saline. Mice were assessed for mechanical, heat, and cold sensitivity. A single behavioral test was performed following each injection to avoid stress-induced effects on testing because each mouse was tested at 1-, 2-, 4-, 6-, and 24-h post-injection.

### Immunofluorescence

Lumbar DRGs 1–6 were isolated from naïve, control, and MIA-injected mice, sectioned on a cryostat at 12 μm thickness for double immunofluorescence labeling (Mishra et al., 2020). Primary antibodies were diluted (1:500 each) in a 5% blocking solution. The primary antibodies used were Tuj1 (Abcam; Cat: ab7751) and GFRα3 (Neuromics, Cat: GT15123). Alexa Fluor conjugated secondary antibodies (Invitrogen; 488, Cat: A20181; 546, Cat: A20183) were applied in a 2% blocking solution for 1 h. Sections were washed, dried, and finalized with mounting media containing DAPI and imaged using a Leica DM5000B microscope at the same exposure setting. Counting was performed on the images by an individual who was blinded to group allocation. A total of 10–13 lumbar DRG sections were counted for each ipsilateral MIA or saline-injected and contralateral control side. DRG images were analyzed for the total number of neurons (Tuj1-positive) and the total number of neurons expressing GFRα3 (Tuj1- and GFRα3-positive). A DRG neuron was deemed positive for GFRα3 or Tuj1 if the staining covered the entire cell body surface and if the intensity value was above the background level as determined by the Region of Interest macro tool in the ImageJ software. A total of 5198, 5193, 1438, and 2315 GFRα3 positive neurons were counted for the MIA contralateral, MIA ipsilateral, saline contralateral, and saline ipsilateral sides, respectively. A total of 27,649, 12,691, 8307, and 11,867 Tuj1 positive neurons were counted for the MIA contralateral, MIA ipsilateral, saline contralateral, and saline ipsilateral sides, respectively.

### Assessment of the Anti-hypersensitivity Effects of a Systemic Anti-artemin Monoclonal Antibody

The anti-hypersensitivity effects of blocking artemin were determined using mice with MIA-induced OA-pain. Following baseline behavioral tests (mechanical, heat, and cold sensitivity testing) mice had OA induced using intra-articular MIA (as described above). Behavioral tests were performed every week up to day 28. Following day 28, mice received a 100 μl intraperitoneal injection of either phosphate buffered saline (PBS) or 25 μg of anti-artemin mAb (R&D, Cat: MAB10851-500). Allocation to treatment was randomized, and testing was performed by an individual blind to treatment allocation. Mice were tested for sensitivity to mechanical, heat, and cold stimuli at 2-, 4-, 6-, and 24-h post-intraperitoneal injection.

### Assessment of the Analgesic Effects of a Systemic Anti-artemin Monoclonal Antibody

In a separate cohort of male and female mice, the analgesic effects of blocking artemin were determined using mice with MIA-induced OA-pain. MIA was injected into the ipsilateral right stifle joint on day 0 to induce OA-pain as described above. A group of control mice received sterile 0.9% saline as described above. Static limb use was assessed at baseline prior to MIA injection, day 14 and day 28 post-injection to validate ipsilateral MIA-induced limb asymmetry, and at 2, 5, and 24-h post-anti-artemin mAb (1 mg/kg in 100 μl PBS, MAB10851 R&D Systems) or anti-IgG isotype control (1 mg/kg in 100 μl PBS, MAB006 R&D Systems) injection. Limb use was measured every 24-h until it returned to 1 standard deviation (SD) of the day 28 post-MIA injection values.

### Experimental Design and Statistical Analysis

All behavioral data were collected by a researcher blind to the groups. Blinding was achieved by having SKM or BDXL prepare syringes of injectate for injection (MIA, saline, anti-artemin mAb, anti-IgG mAb) and concealing the identity of the contents from the individual performing the behavioral assessments.

Mice for experiments were selected based on availability of mice from the breeding program. Once selected for an experiment, mice were randomly assigned to experimental groups using an online randomizer.^1^ All mice assigned to an experimental group were included in data analysis—no mice were excluded. The majority of these experiments reported here used only male mice to avoid the effects of the estrus cycle, but future work should use both sexes. Both male and female mice were used to test the analgesic effects of the anti-artemin mAb on limb use in mice with MIA-induced OA-pain.

Sample sizes were based on the following data generated in pilot work. For testing hypersensitivity in response to MIA-induced OA-pain, pilot work indicated a delta of 6.0 g at 28 days, with a pooled SD of 1.9 g, indicating that four mice per group would provide 90% power. For testing the change in hypersensitivity in MIA mice in response the anti-artemin mAb, pilot work showed a delta of 3.7 g between groups at 28 days, with a pooled SD of 2.1 g, indicating that eight mice per group would provide 90% power. In the limb use experiments, pilot work indicated that at day 28 the difference in limb weight-bearing between controls and MIA mice was 1.25 g, with a pooled SD of 0.55, suggesting that six mice per group would provide 90% power. There were no pilot data available for the experiment to assess hypersensitivity associated with artemin, and so the largest group size required in other experiments (*n* = 8) was used. Pilot data using anti-artemin mAb in mice with MIA-induced OA-pain showed a weight symmetry index difference between the treatment and control of 30, and a pooled SD of 8, suggesting three mice per group would give 90% power.

**FIGURE 1:**
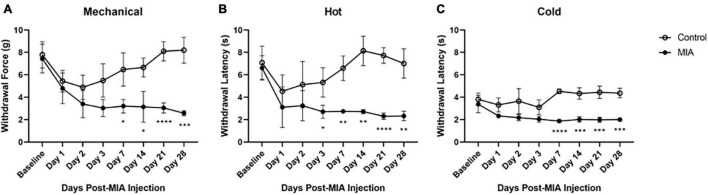
Hypersensitivity to mechanical, heat, and cold stimuli after MIA induction. Mice were tested for hypersensitivity to mechanical **(A)**, heat **(B)**, and cold **(C)** stimuli using the von Frey, Hargreaves, and crushed dry ice assays over 28 days following MIA injection. MIA (*n* = 5) and control (*n* = 6). Data are represented as mean ± SD (*p < 0.05; **p < 0.01; ***p < 0.001; ****p < 0.0001). Two-way ANOVA with repeated measures and Geisser–Greenhouse and Sidak corrections.

**FIGURE 2:**
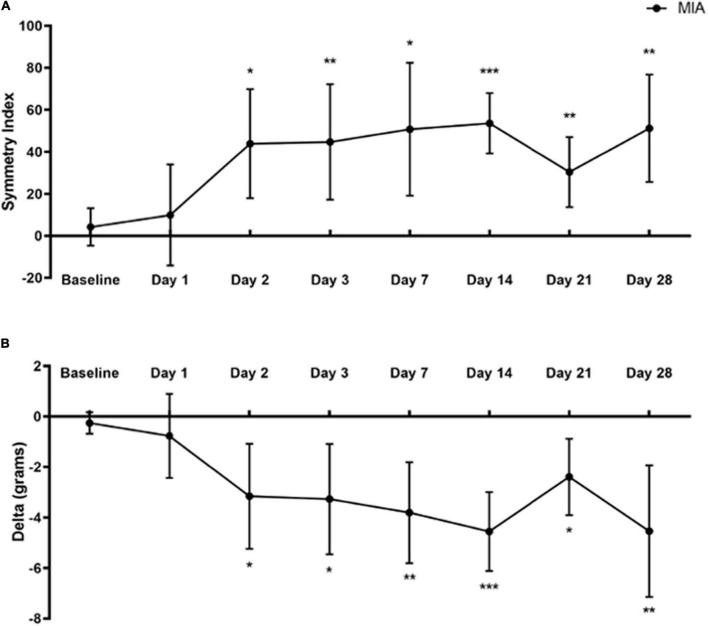
Static weight-bearing after MIA induction. Standing body weight distribution on the hind limbs was measured over 28 days following intra-articular MIA injection in mice. Hind limb Symmetry Index **(A)**, and Delta, or the difference between the weight placed on the MIA-injected right and control left legs **(B)** was determined at baseline, 1, 2, 3, 7, 14, 21, and 28-days post-MIA injection. Mice displayed a greater degree of MIA-induced asymmetry and decreased weight-bearing on day 2 and onward, compared with the pre-injection baseline values. MIA (*n* = 8). Data are represented as mean ± SD (*p < 0.05; **p < 0.01; ***p < 0.001). A one-way ANOVA with repeated measures and Geisser–Greenhouse and Dunnett multiple corrections.

**FIGURE 3:**
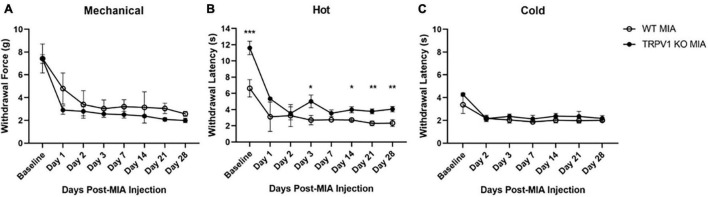
Monoiodoacetate-induced hypersensitivity in *Trpv1* KO mice. Mechanical **(A)**, heat **(B)**, and cold **(C)** sensitivity were measured at baseline and over 28 days following MIA induction of OA-pain in wild-type (WT) and *Trpv1* knockout (KO) mice. **(A,C)** There was no statistically significant difference between WT and *Trpv1* KO mice at baseline or at any time points post-MIA injection. **(B)** *Trpv1* KO mice had significantly longer withdrawal latencies (decreased sensitivity) to a heat stimulus at baseline, days 3, 14, 21, and 28 post-MIA than WT mice. WT MIA (*n* = 5), *Trpv1* KO MIA (*n* = 4). Data are represented as mean ± SD (*p < 0.05; **p < 0.01; ***p < 0.001). Two-way ANOVA with repeated measures and Geisser–Greenhouse and Sidak corrections.

**FIGURE 4:**
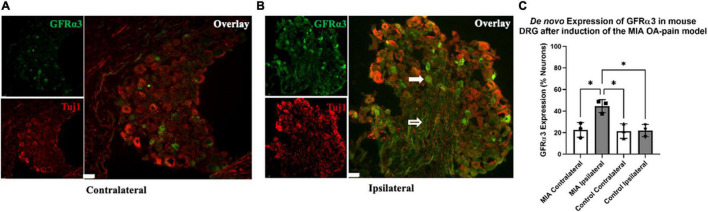
*De novo* expression of GFRα3 in MIA-induced mouse model of OA-pain. Contralateral **(A)** and ipsilateral **(B)** mouse lumbar 1–6 DRG were double immunostained with Tuj1 in red and GFRα3 in green. The white filled arrow highlights a neuronal cell body that is positive for both Tuj1 and GFRα3. The white unfilled arrow points to a nerve afferent that is positive for GFRα3. The white marker bar represents 75 μm. **(C)** Quantification of GFRα3 normalized against a pan-neuronal marker (Tuj1) revealed an increase in GFRα3 in mice injected with MIA. A total 10–13 sections from each side were counted for the number of neurons (Tuj1) and the number of neurons expressing GFRα3. We found a significant difference in GFRα3 expression between the MIA ipsilateral and MIA contralateral, saline ipsilateral, and saline contralateral lumbar DRG’s. Control (*n* = 3) and MIA (*n* = 3). Data are represented as mean ± SD (*p < 0.05). An ordinary one-way ANOVA with Holm-Sidak multiple comparisons test.

**FIGURE 5:**
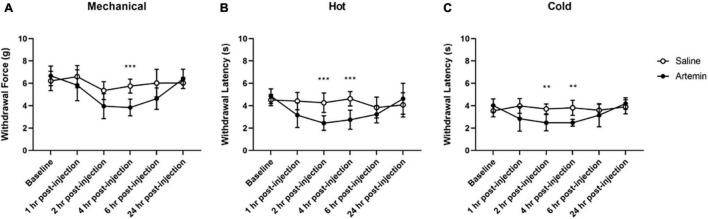
Artemin-induced hypersensitivity to mechanical, heat, and cold stimuli. Mice that received artemin had significantly reduced withdrawal latencies to mechanical **(A)**, heat **(B)**, and cold **(C)** compared to mice that received saline at 4-h post-injection. Withdrawal latencies to the hot and cold stimuli were also significantly lower at 2-h post-artemin injection, compared with the saline-injected group. Data are represented as mean ± SD (**p < 0.01; ***p < 0.001). For mechanical hypersensitivity artemin (*n* = 8) and saline (*n* = 8). For thermal hypersensitivity artemin (*n* = 9) and saline (*n* = 9). Two-way ANOVA with repeated measures and Geisser–Greenhouse and Sidak corrections.

**FIGURE 6:**
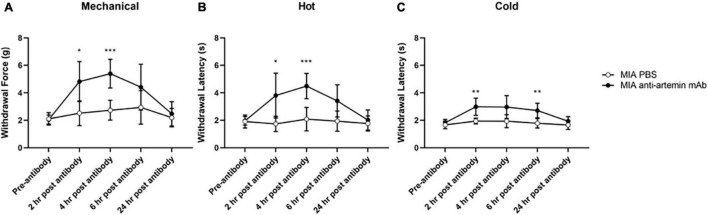
An anti-artemin antibody alleviates hyperalgesia in the MIA-induced mouse model of OA-pain. A systemic anti-artemin monoclonal antibody alleviates MIA-associated hypersensitivity for mechanical **(A)**, heat **(B)**, and cold **(C)**. Anti-artemin mAb was effective in reversing mechanical and heat hypersensitivity at 2- and 4-h after antibody. Anti-artemin mAb reversed cold hypersensitivity at 2 and 4 h post-mAb injection, compared with PBS injected MIA controls. MIA anti-artemin mAb (*n* = 8) and MIA PBS (*n* = 7). Data are represented as mean ± SD (*p < 0.05; **p < 0.01; ***p < 0.001). Two-way ANOVA with repeated measures and Geisser–Greenhouse and Sidak corrections.

**FIGURE 7:**
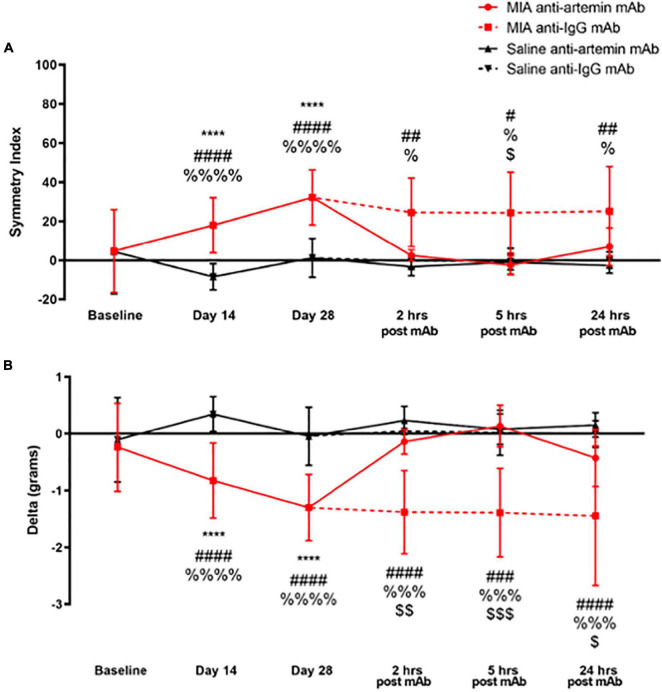
An anti-artemin antibody rescues MIA-induced limb asymmetry. Hind limb Symmetry Index **(A)**, and Delta or the difference between the weight placed on the MIA-injected right and control left legs **(B)** was determined at baseline, days 14, 28 and 2-, 5-, and 24-h after systemic anti-artemin or anti-IgG monoclonal antibody administration. The anti-artemin antibody reversed MIA-induced limb asymmetry and returned Delta to baseline values at 2-, 5-, and 24-h post-antibody administration compared with the isotype control group. Asterisk compares MIA/anti-artemin and saline anti-artemin and anti-IgG groups, ^#^compares MIA/anti-IgG and saline/anti-artemin, ^%^compares MIA/anti-IgG and saline/anti-IgG, ^$^compares MIA/anti-artemin and MIA/anti-IgG groups. Baseline (MIA males *n* = 8, MIA females *n* = 0, saline males *n* = 8, saline females *n* = 0), day 14 (MIA males *n* = 7, MIA females *n* = 5, saline males *n* = 8, saline females *n* = 7), day 28 (MIA males *n* = 7, MIA females *n* = 5, saline males *n* = 8, saline females *n* = 6), 2–24 h (MIA anti-artemin males *n* = 3, MIA anti-artemin females *n* = 2, MIA anti-IgG males *n* = 2, MIA anti-IgG females *n* = 2, saline anti-artemin males *n* = 3, saline anti-artemin females *n* = 4, saline anti-IgG males *n* = 3, saline anti-IgG females *n* = 3). Data are represented as mean ± SD (^#^, ^%^, and ^$^*p* < 0.05; ^##^ and ^$$^*p* < 0.01; ^###^, ^%%%^, and ^$$$^*p* < 0.001; ****, ^####^, and ^%%%%^*p* < 0.0001). Two-way ANOVA with repeated measures and Holm-Sidak multiple comparisons tests.

We used JMP Pro 14.1 for Mac (2018 SAS Institute Inc., Raleigh, NC, United States), GraphPad Prism 6.0c for Mac (GraphPad Software, La Jolla, CA, United States), and ImageJ (Schneider et al., 2012) for data manipulation, statistical analyses, and for generating figures. Data were tested for normalcy with the Shapiro–Wilk test. A repeated measures one-way ANOVA with Geisser–Greenhouse and Dunnett multiple corrections tests was used to evaluate the changes in the mean difference for weight-bearing measures over multiple time points (Figure 2). An ordinary one-way ANOVA with Holm-Sidak multiple comparisons (Figure 4) or two-way repeated measures ANOVA with Sidak corrections (Figures 1, 3, 5, 6, 7) were performed to evaluate the mean differences between groups with two or more independent variables. The assumptions of homoscedasticity and normal distribution of residuals were checked. Statistical significance was determined using a corrected *p*-value of 0.05. All statistical tests are described in the results and figure legends.

## Results

### Monoiodoacetate-Induced Osteoarthritis-Pain Is Associated With Mechanical, Heat, and Cold Hypersensitivity

A two-way ANOVA with repeated measures and Geisser–Greenhouse and Sidak corrections was used to evaluate MIA-induced changes in mechanical, heat, and cold hypersensitivity after intra-articular injection with either MIA (1 mg/10 μl) or saline (10 μl). Mice were tested at 1-, 2-, 3-, 7-, 14-, 21-, and 28-days following injection of MIA. The sensitivity of the contralateral limb of the MIA-injected mice did not differ from the contralateral or ipsilateral limbs of the saline-injected mice (data not shown). The ipsilateral MIA-injected hind limb was hypersensitive to mechanical, hot, and cold stimuli, compared to the ipsilateral limb of the saline-injected controls.

For mechanical hypersensitivity there was a statistically significant effect of time [*F*(4.125,37.12) = 15.43, *p* < 0.0001], group [*F*(1,9) = 45.87, *p* < 0.0001], and an interaction between group and time points [*F*(7,63) = 12.32, *p* < 0.0001]. There was a significant difference between the MIA and saline-injected groups at 7 (*p* = 0.141), 14 (*p* = 0.161), 21 (*p* < 0.001), and 28 (*p* < 0.001) days post-MIA injection (Figure 1A). For hypersensitivity to a hot stimulus there was a statistically significant effect of time [*F*(3.189,28.70) = 6.779, *p* = 0.001], group [*F*(1,9) = 228.3, *p* < 0.0001], and an interaction between group and time points [*F*(7,63) = 6.431, *p* < 0.001]. There was a significant difference between the MIA and saline-injected groups at 3 (*p* = 0.023), 7 (*p* = 0.002), 14 (*p* = 0.001), 21 (*p* < 0.0001), and 28 (*p* = 0.001) days post-MIA injection (Figure 1B). For cold hypersensitivity there was a statistically significant effect of time [*F*(3.994,35.95) = 5.624, *p* = 0.001], group [*F*(1,9) = 74.10, *p* < 0.001), and an interaction between group and time points [*F*(7,63) = 10.20, *p* < 0.001]. There was a significant difference between the two groups at 7 (*p* < 0.001), 14 (*p* = 0.001), 21 (*p* = 0.0002), and 28 (*p* = 0.002) days post-MIA injection (Figure 1C). For all mechanical and thermal assays, the MIA-injected mice showed progressive lameness over the course of the 28 days.

### Monoiodoacetate-Induced Osteoarthritis-Pain Reduces Body Weight Distribution to the Painful Limb

A one-way repeated measures ANOVA with Geisser–Greenhouse and Dunnett multiple corrections test examined the effects of MIA induced changes in weight-bearing at baseline and up to 28 days post-injection on SI and Delta values using the SHIM (Williams et al., 2019). Mice placed significantly less weight on the MIA-injected limb than the contralateral control limb starting at day 2 and continuing to day 28 post-MIA injection.

For SI, there was a statistically significant difference between baseline and post-MIA injection time points [*F*(4.199,29.39) = 7.421, *p* = 0.0002]. There was a significant increase in the degree of asymmetry at 2 (*p* = 0.014), 3 (*p* = 0.008), 7 (*p* = 0.011), 14 (*p* = 0.001), 21 (*p* = 0.007), and 28 days (*p* = 0.002) post-MIA injection, compared with baseline (Figure 2A).

For the Delta between limbs, there was a statistically significant difference between baseline and post-MIA injection time points [*F*(4.568,31.97) = 8.905, *p* < 0.0001]. Compared with pre-injection baseline values, MIA injection into the ipsilateral right limb resulted in decreased weight-bearing on the right limb at 2 (*p* = 0.023), 3 (*p* = 0.015), 7 (*p* = 0.004), 14 (*p* = 0.001), 21 (*p* = 0.014), and 28 days (*p* = 0.008) (Figure 2B).

### Role of Transient Receptor Potential Vanilloid Subfamily-1 in Monoiodoacetate-Induced Osteoarthritis Hypersensitivity

TRP channels are expressed in peripheral afferents and play a role in directly detecting noxious heat stimuli. To determine the contribution of TRPV1 in MIA-induced OA-pain, we used *Trpv1* KO (Caterina et al., 1999) mice and compared responses to control wild-type littermates. We used a two-way ANOVA with repeated measures and Geisser–Greenhouse and Sidak corrections for all comparisons. For the noxious heat sensitivity assay there was a statistically significant interaction within time points [*F*(2.004,14.03) = 61.62, *p* < 0.0001], groups [*F*(1,7) = 76.89, *p* < 0.0001], and an interaction between groups and time points [*F*(7,49) = 7.556, *p* < 0.0001]. *Trpv1* KO mice had an increased withdrawal latency with the Hargreaves assay compared to wild-type mice at baseline (*p* < 0.001) and post-MIA at days 3 (*p* = 0.028), 14 (*p* = 0.024), 21 (*p* = 0.002), and 28 (*p* = 0.002); i.e., the *Trpv1* KO mice were less sensitive to heat, as expected (Figure 3B).

The *Trpv1* KO status had no effect on mechanical and cold hypersensitivity induced by MIA (Figures 3A,C). For the mechanical sensitivity assay there was a statistically significant effect of time [*F*(2.559,17.91) = 52.14, *p* < 0.0001] and group [*F*(1,7) = 6.405, *p* = 0.039]. There was no significant interaction between time points and group [*F*(7,49) = 1.320, *p* = 0.261]. For the cold sensitivity assay there was a statistically significant of time [*F*(2.865,18.79) = 38.36, *p* < 0.0001] and group [*F*(1,7) = 11.71, *p* = 0.011]. There was no significant interaction between time points and groups [*F*(6,42) = 1.863, *p* = 0.110]. No data were collected at day 1 post-MIA for the cold sensitivity assay.

### GFRα3 Receptor Expression Is Increased in Monoiodoacetate-Induced Osteoarthritis-Pain

Recently, we showed an increase in the expression of GFRα3 (gene and protein) in the DRG of dogs with naturally occurring OA (Minnema et al., 2020). However, it was unclear if some of the increase in GFRα3 expression was due to *de novo* increase in the number of GFRα3-positive neurons. To determine whether there is either an increase in protein or an increase in the number of GFRα3-positive neurons, we performed double immunofluorescence staining in contralateral and ipsilateral lumbar 1–6 DRGs of MIA and saline-injected mice. The images in Figures 4A,B highlight a lumbar DRG cross-section where both the neuronal cell bodies and nerve afferents are visible. The white filled arrow highlights a neuronal cell body that is positive for both Tuj1 and GFRα3. The white unfilled arrow points to a nerve afferent that is positive for GFRα3. An ordinary one-way ANOVA was performed to compare the GFRα3-positive neurons between groups. It revealed a statistically significant difference in the number of GFRα3-positive cells between groups [*F*(3,8) = 9.465, *p* = 0.005]. A Holm-Sidak multiple comparisons test showed a significant difference in GFRα3 expression between the MIA ipsilateral and MIA contralateral (*p* = 0.013), control contralateral (*p* = 0.013) and contralateral (*p* = 0.013) lumbar DRG’s. Overall, we found that GFRα3 was expressed in ∼ 44% of DRG neurons serving MIA-injected joints compared to ∼22% of neurons serving the contralateral joints and DRG serving joints that received saline (Figures 4A–C).

### Artemin, a Ligand of GFRα3 Receptor, Plays a Role in Thermal and Mechanical Localized Hypersensitivity

Osteoarthritis is associated with mechanical and thermal hypersensitivity. Previously, we determined that artemin, the ligand for GFRα3, is increased in the synovial fluid and serum of dogs and the serum of humans with naturally occurring OA. Therefore, we sought to determine if artemin is associated with mechanical and thermal peripheral localized hypersensitivity. We used a two-way ANOVA with repeated measures and Geisser–Greenhouse and Sidak corrections for all comparisons.

For mechanical hypersensitivity we found a significant effect of time [*F*(3.687,51.62) = 10.91, *p* < 0.0001], group [*F*(1,14) = 17.63, *p* = 0.0009], and a significant interaction between group and time points [*F*(5,70) = 4.333, *p* = 0.0017]. We found that injection of artemin into the plantar surface of the paw was associated with significantly increased mechanical hypersensitivity at 4-h post-injection (*p* < 0.001, Figure 5A). For sensitivity to a noxious heat stimulus measured using the Hargreaves assay we found a significant effect of time [*F*(3.425,54.80) = 7.981, *p* < 0.0001], group [*F*(1,16) = 9.382, *p* = 0.0074], and a significant interaction between group and time points [*F*(5,80) = 8.515, *p* < 0.0001]. A Sidak multiple comparisons test revealed a significant difference between the artemin and saline-injected groups at 2- (*p* = 0.001) and 4-h (*p* < 0.001) post-injection (Figure 5B). For the dry ice (cold) assay, there was a significant interaction effect of time [*F*(2.885,46.15) = 5.214, *p* = 0.0039], group [*F*(1,16) = 20.34, *p* = 0.0004], and a significant interaction between group and time points [*F*(5,80) = 6.047, *p* < 0.0001] for the dry ice assay. *Post hoc* testing revealed a significant difference between the artemin and saline-injected groups at 2- (*p* = 0.004) and 4-h (*p* = 0.001) post-injection (Figure 5C). All mice returned to baseline by 24-h post-injection.

### Blocking Signaling Between Artemin and GFRα3 Attenuates Mechanical and Thermal Hypersensitivity in Monoiodoacetate-Induced Osteoarthritis-Pain

Monoiodoacetate injected into the right stifle joint was used to induce OA-pain as described earlier. Mice were tested weekly for 28 days to confirm hypersensitivity (data not shown). Anti-artemin mAb (25 μg/100 μl; intraperitoneal) was administered on day 28. We used vehicle (1× PBS) as a diluent control for mAb injection. A two-way ANOVA with repeated measures and Sidak corrections examined the effects of the anti-artemin or PBS control injection at 2-, 4-, 6-, and 24-h post-injection on mechanical and thermal hypersensitivity.

For mechanical hypersensitivity there was a significant effect of time [*F*(2.915,37.89) = 13.56, *p* < 0.0001], groups [*F*(1,13) = 18.39, *p* = 0.0009], and a significant interaction between group and time points [*F*(4,52) = 6.097, *p* = 0.0004]. Mice that received the anti-artemin antibody resulted in increased mechanical thresholds compared to PBS-injected MIA controls between 2- (*p* = 0.015) and 4-h (*p* < 0.001) post-injection (Figure 6A). The anti-artemin injected group also had a higher withdrawal latency at 2- (*p* = 0.042) and 4-h (*p* < 0.001) post-mAb injection for heat sensitivity and 2- (*p* = 0.009) and 6-h (*p* = 0.008) post-mAb injection for cold sensitivity, compared with the control MIA group (Figures 6B,C). All mice were back to pre-injection response levels by 24-h of post-antibody injection.

### Blocking Signaling Between Artemin and GFRα3 Returns Limb Use to Normal in Monoiodoacetate-Induced Osteoarthritis-Pain

A two-way ANOVA with repeated measures and Sidak corrections examined the effects of MIA or saline injection at baseline, days 14 and 28, and anti-artemin or anti-IgG mAb injection at 2-, 5-, and 24-h post-injection on SI and Delta values.

As illustrated in Figure 7A, there was a statistically significant effect of time [*F*(5,180) = 6.090, *p* < 0.001], groups [*F*(3,180) = 27.88, *p* < 0.001], and a significant interaction between groups and time points [*F*(15,180) = 3.286, *p* < 0.001] for SI. As a result of OA-induced pain, the degree of asymmetry between the MIA-injected ipsilateral limb and the non-injected contralateral control leg significantly increased on day 14 (*p* < 0.001) and 28 (*p* < 0.001), compared with the saline-injected group and baseline values. The MIA-injected mice that received the anti-artemin mAb significantly improved limb use compared with the isotype control group, and SI returned to baseline values at 2-, 5-, and 24-h. post-injection. The MIA/anti-IgG isotype control group continued to have increased limb asymmetry similar to days 14 and 28. There was a significant difference between the MIA/anti-IgG group and the saline/anti-artemin and saline/anti-IgG groups at 2- (*p* = 0.005, *p* = 0.020), 5- (*p* = 0.013, *p* = 0.020), and 24-h (*p* = 0.005, *p* = 0.020). The SI of the MIA/anti-artemin group was significantly lower (less asymmetry) compared with the MIA/anti-IgG group at 5-h. post-injection (*p* = 0.01). There was no change in SI for the saline/anti-artemin or saline/anti-IgG groups. The SI values for the anti-artemin mAb injected MIA group returned to day 28 post-MIA injection baseline values 3–5 days after mAb injection in all mice.

For the difference between the weight placed on the right and left legs (Delta in grams), there was a statistically significant effect of time [*F*(5,180) = 4.750, *p* < 0.001], groups [*F*(3,180) = 40.80, *p* < 0.001], and an interaction between groups and time points [*F*(15,180) = 3.445, *p* < 0.001] (Figure 7B). Compared with pre-injection baseline values, MIA injection into the ipsilateral right limb resulted in decreased weight-bearing on the right limb on days 14 (*p* < 0.0001) and 28 (*p* < 0.0001) compared to the saline-injected group. A systemic (intraperitoneal) anti-artemin mAb injection reversed the ipsilateral limb disuse compared with the anti-IgG isotype control in the MIA group at 2- (*p* = 0.006), 5- (*p* < 0.001), and 24-h (*p* = 0.038) post-injection. Limb use in the MIA anti-artemin mAb group returned to pre-MIA injection baseline values. There was no statistically significant difference between the MIA anti-artemin mAb and the saline mAb groups at 2-, 5-, and 24-h. Delta values for the MIA anti-artemin mAb group returned to day 28 post-MIA injection values 3–5 days after the mAb injection in all mice.

## Discussion

Here, using a mouse model of OA and OA-pain, and clinically relevant measures of sensitivity and limb use, we explored the functional role of artemin/GFRα3 signaling in OA-pain. We found the MIA-induced OA-pain mouse model is associated with hypersensitivity and decreased limb use. *Trpv1* KO mice were partially protected against heat hypersensitivity, suggesting the involvement of other TRP channels in heat hypersensitivity in OA-pain. Yet, there was no effect of the *Trpv1* KO status on mechanical and cold related sensitivities. We demonstrated that in MIA-induced OA-pain, GFRα3 expression is increased in sensory neurons. Further, exogenous artemin induces mechanical and thermal hypersensitivity, and anti-artemin mAb administration reverses MIA-induced evoked pain hypersensitivity and limb disuse. Overall, our results indicate artemin/GFRα3 plays a functional role in OA-pain.

### Mouse Monoiodoacetate Model of Osteoarthritis-Pain

Mechanisms of OA-pain have been widely studied in rat and mouse models employing intra-articular injection of chondrocyte glycolytic inhibitor MIA (Bandell et al., 2004; Fernihough et al., 2004; Bautista et al., 2007; Dhaka et al., 2007; Cavanaugh et al., 2009; Malfait et al., 2013; Sousa-Valente et al., 2018). Many groups have demonstrated mechanical hypersensitivity and changes in limb use (Harvey and Dickenson, 2009; Ogbonna et al., 2013; Kuyinu et al., 2016; Yuan et al., 2018). However, mixed results have been obtained with respect to changes in hypersensitivity in MIA-injected mice (Harvey and Dickenson, 2009; Yuan et al., 2018). Thus, our goal was to characterize changes in hypersensitivity to mechanical, hot, and cold stimuli and static limb use all in the same sets of experiments. We utilized a 1 mg MIA dose based on the literature focusing on OA-associated mechanical hypersensitivity and changes in weight distribution, and our own pilot data. In this study we showed increased sensitivity to mechanical, hot, and cold stimuli in mice with MIA-induced OA-pain compared to controls. These changes in mechanical and thermal sensitivities mirror what is seen in humans with OA-pain (Fingleton et al., 2015) and dogs with naturally occurring OA-pain (Freire et al., 2016; Knazovicky et al., 2016). Approximately 70% of human patients with knee OA experience hypersensitivity to at least one mechanical or thermal modality (Wylde et al., 2011a,b, 2012a,2012b). In a separate experiment, we measured body weight distribution using incapacitance meters and found a reduction in limb use in the MIA-injected mice, suggesting ongoing joint pain. Human knee OA patients also display weight-bearing asymmetry while at rest with less weight born on the affected limb (Christiansen and Stevens-Lapsley, 2010; Duffell et al., 2013; Harato et al., 2014), again demonstrating the relevance of the murine MIA model of OA-pain. To the best of our knowledge, no other laboratories have confirmed the combination of mechanical, heat, and cold hypersensitivity and decreased limb use in the MIA-induced mouse model of OA-pain.

### Role of Transient Receptor Potential Vanilloid Subfamily-1 Ion Channel in Osteoarthritis-Pain

The TRPV1 receptor has been widely studied for its role in the transduction of stimuli and the generation of pain induced by noxious heat (Caterina et al., 1999; Cavanaugh et al., 2009). Recently, the role of TRPV1 in pain and sensitivity associated with OA-pain has been reported across various models and species. In one study in MIA-injected rats, a TRPV1 antagonist blocked the development of heat hypersensitivity, but not weight-bearing deficits or ongoing pain (Okun et al., 2012). In a complete Freund’s Adjuvant (CFA) stifle OA model in mice, the TRPV1 antagonist, A-425619, reversed the decrease in digging behavior induced by the CFA injection (Chakrabarti et al., 2018). There are no reports of the role of TRPV1 in the murine MIA model of OA-pain. TRPV1 antagonism has only shown mild therapeutic effects in humans (Mayorga et al., 2017) and a high incidence of adverse events; and in dogs with naturally occurring OA-pain, a TRPV1 antagonist was not effective (Malek et al., 2012). In our study, we used *Trpv1* KO mice to examine if TRPV1 is involved in MIA-induced mechanical and thermal hypersensitivity. Not to our surprise, we found the loss of function of TRPV1 was partially protective for heat sensitivity induced by MIA at day 28, but *Trpv1* KO did not affect mechanical and cold hypersensitivity. The role of TRPV1 in detecting heat but not mechanical sensation is consistent with previous reports (Cavanaugh et al., 2009; Mishra et al., 2020). Collectively, the literature and our results indicate little evidence of an analgesic effect of blocking or removing the influence of the TRPV1 receptor, despite its known role in pain processes. These findings suggest that both TRPV1 dependent and TRPV1 independent components may be involved in sensing peripheral sensitivity to a noxious hot stimulus. Thus, other TRP channels (TRPM3, TRPV2, TRPV3, TRPV4) and signaling systems may be involved in OA-pain sensation, and upstream signaling mechanisms may be more effective therapeutic targets.

### Monoiodoacetate-Induced *de novo* Expression of GFRα3 in Murine Dorsal Root Ganglia

Recently, we identified an increase in the expression of GFRα3 at both the protein and RNA levels in the naturally occurring dog model of OA-pain, suggesting this change in the GFRα3 protein is due to underlying disease conditions. The role of GFRα3 in pain has been partially established through work showing GFRα3 signaling in cold pain (Lippoldt et al., 2013), bladder pain (DeBerry et al., 2015), and inflammatory bone pain (Nencini et al., 2018). However, the role of GFRα3 signaling in arthritis pain has not been investigated to date. Interestingly, our data suggest a *de novo* expression of GFRα3 in the DRG, doubling the number of neurons expressing the GFRα3 receptor, perhaps indicating a change in neuronal plasticity of sensory neurons. Here, we evaluated lumbar DRG (L1–L6) instead of only focusing on neural afferents that directly serve the stifle joint, which is a limitation of our work. Our future work should characterize changes in the GFRα3 neuronal population in the naïve and MIA-induced OA-pain state at both the RNA and protein level on the DRG cell bodies that directly serve the stifle joint.

### Role of Artemin/GFRα3 Signaling in Osteoarthritis-Pain

Osteoarthritis is a degenerative disease, and in the process of tissue degeneration, various mediators are released. Based on our data in naturally occurring osteoarthritic dogs, and confirmed in a small number of human samples, we found artemin was increased in the serum in association with OA-pain, and synovial fluid artemin concentrations were associated with joint pain (as measured by decreased limb kinetic variables) (Minnema et al., 2020). Further, increased serum artemin was associated with higher total joint pain scores in dogs with OA-pain. Interestingly, there was no significant relationship between higher serum artemin and widespread sensitivity (Gupta et al., 2021). This result does not negate the role of artemin in the establishment or maintenance of OA-pain and associated sensitivity, as this work was within a population of dogs with OA-pain and was focused on widespread OA-associated sensitivity, not localized sensitivity.

Data from our present study shows that a single local injection of artemin into a mouse’s hind paw can induce short-term localized peripheral hypersensitivity to mechanical and thermal stimuli, which are also features of human OA patients. Other research groups have also investigated if peripherally administered artemin induces hypersensitivity, but there is no clear consensus across groups. Per Ikeda-Miyagawa et al. (2015), a one-time artemin injection did not induce any hypersensitivity measured at 1–5 days post-injection, but repeated artemin application increased mechanical and heat hypersensitivity (Ikeda-Miyagawa et al., 2015). In contrast, we noted a short-term effect of hypersensitivity at 2- to 4-h post-injection—this effect of artemin injection may have been missed by Ikeda-Miyagawa et al. (2015) due to the time points evaluated. Intraplantar injection of artemin induces mechanical and heat hyperalgesia within 4-h post-injection (Thornton et al., 2013), which is in agreement with our findings. However, in another study, investigators found no change in response to mechanical stimuli post-artemin injection (Lippoldt et al., 2013). Interestingly, heat and cold hypersensitivity in response to artemin injection were observed for 1- to 3-h post-injection (Lippoldt et al., 2013). We found mechanical hypersensitivity in addition to heat and cold hypersensitivity post-artemin injection. The studies by Lippoldt et al., 2013 used., both sexes of mice. A limitation of our study is that we used only male mice. This may explain some of the differences we see for mechanical and heat hypersensitivity. We only used males in our earlier work to avoid the impact of the estrous cycle on pain sensitivity (Wiesenfeld-Hallin, 2005), but as we saw positive results, we moved to include both sexes. Future experiments should use both sexes.

The expression of GFRα3 in naïve DRG and its co-expression and interaction with nociceptive channels is known, but its role in chronic pain states is yet to be explored. Reports have shown that artemin caused an increase in the mRNA for GFRα3, tropomyosin receptor kinase A (TrkA), TRPV1, and TRPA1 (Elitt et al., 2006), and an anti-artemin antibody was shown to block upregulation of TRPA1 (DeBerry et al., 2015), and knockdown of GFRα3 with siRNA blocked the upregulation of TRPV1 in a nerve injury model (Jankowski et al., 2010). The difference between the effect of the *Trpv1* KO status and mAb anti-artemin suggests that blocking artemin/GFRα3 signaling may have more analgesic potential than targeting individual TRP receptors because GFRα3 may be acting upstream of these nociceptive TRP channels.

Here, we demonstrate that blocking the signaling between artemin/GFRα3 by a mAb sequestration of artemin in the MIA mouse model of OA-pain inhibits mechanical, heat, and cold sensitivity and returns limb use to normal. Since these antibodies cannot cross the blood-brain barrier (Pardridge, 2019), these findings support the notion that peripheral sequestration of artemin is responsible for maintaining pain in the MIA model.

In our limb use data set at 5-h post-mAb injection, the MIA anti-artemin mAB group had a standardized effect size of 1.78 compared with the MIA IgG group. However, it is important to compare the effect size of anti-artemin mAb with other analgesic treatments to understand the efficacy of targeting the artemin/GFRα3 pathway. A literature review showed that a similar reversal in the difference in weight-bearing (grams) was demonstrated in the intra-articular carrageenan model of inflammatory pain in rats with a twice-daily oral dose of a selective TrkA inhibitor on days 1, 2, and 4 (Ashraf et al., 2016). In this study, the TrkA inhibitor had an effect size of 1.50 on day 1 compared with rats that received the vehicle treatment.

In Figures 6, 7, we noted an effect of the anti-artemin mAb for up to 6-h for relieving MIA-induced hypersensitivity, and up to 3–5 days for limb use. We presented data for up to 24-h in both figures post-mAb administration. This somewhat transient effect may reflect our use of a one-time low dose of the anti-artemin mAb (25 μg in 100 μl of sterile 1× PBS at a final dose of 1 mg/kg, R&D, Cat: MAB10851). Edamitsu et al. (2019) also used MAB10851 and found a reduction in scratching behavior that lasted for up to 11 weeks, but after administering the mAb once per week for 11 weeks and at a dose of 50 mg/kg, which is fifty times our dose. Lippoldt et al. (2016) also utilized MAB10851 to show that artemin neutralization selectively attenuated cold hypersensitivity in the CFA and oxaliplatin mouse models. They administered a one-time dose of 10 mg/kg (ten times our dose) yet reported a shorter duration of the anti-artemin mAb (4-h) compared to our work. Thornton et al. (2013), using the CFA mouse model of inflammation and a one-time MAB10851 dose of 30 or 10 mg/kg showed a significant improvement in weight distribution at only 4-h post-mAb administration compared to the isotype control. Thus, since we administered a single low dose of 1 mg/kg at only one-time point, we feel confident in our positive results.

Overall, we present the first evidence of a potential functional role of artemin/GFRα3 in chronic OA-pain in mice. However, there is much to understand about the potential role of artemin, including the mechanisms leading to artemin release and which cells types in the joints are responsible for artemin release; whether artemin is involved in the induction and/or maintenance of OA-pain; and whether artemin acts through only GFRα3, or other receptors [such as NCAM (Schmutzler et al., 2011)] are involved. Also, the degree to which artemin may drive the ultimate experience of OA-pain needs to be fully elucidated. Although we have found that artemin’s cognate receptor, GFRα3, was upregulated in MIA-induced OA-pain, paralleling what we found in the naturally occurring OA model in the dog, work needs to be done to determine the contribution of GFRα3 upregulation and/or activation in OA-pain, and further, the downstream targets and signaling mechanisms need to be defined.

## Data Availability Statement

The raw data supporting the conclusions of this article will be made available by the authors, without undue reservation.

## Ethics Statement

The animal study was reviewed and approved by the North Carolina State University IACUC (approval #19-047-B).

## Author Contributions

SM and BDL conceptualized the study and designed the experiments. LM and AG performed the experiments and analyzed the data with SM and BDL. SM, BDL, and AG wrote the manuscript. All authors approved the manuscript.

## Conflict of Interest

The authors declare that the research was conducted in the absence of any commercial or financial relationships that could be construed as a potential conflict of interest.

## Publisher’s Note

All claims expressed in this article are solely those of the authors and do not necessarily represent those of their affiliated organizations, or those of the publisher, the editors and the reviewers. Any product that may be evaluated in this article, or claim that may be made by its manufacturer, is not guaranteed or endorsed by the publisher.

## Footnotes

1
https://www.random.org/lists/
